# Iron, hepcidin, and the metal connection

**DOI:** 10.3389/fphar.2014.00128

**Published:** 2014-06-04

**Authors:** Olivier Loréal, Thibault Cavey, Edouard Bardou-Jacquet, Pascal Guggenbuhl, Martine Ropert, Pierre Brissot

**Affiliations:** ^1^INSERM UMR 991, Iron and the Liver TeamRennes, France; ^2^Faculty of Medicine, University of Rennes1Rennes, France; ^3^CHU Pontchaillou, French Reference Centre for Rare Iron Overload Diseases of Genetic Origin, University Hospital-RennesRennes, France; ^4^Biochemistry and Enzymology Laboratory, Centre Hospitalier UniversitaireRennes, France; ^5^Department of Rheumatology, Centre Hospitalier UniversitaireRennes, France

**Keywords:** iron, metal, metabolism, disease, ferroportin, DMT1, transferrin

## Abstract

Identification of new players in iron metabolism, such as hepcidin, which regulates ferroportin and divalent metal transporter 1 expression, has improved our knowledge of iron metabolism and iron-related diseases. However, from both experimental data and clinical findings, “iron-related proteins” appear to also be involved in the metabolism of other metals, especially divalent cations. Reports have demonstrated that some metals may affect, directly or indirectly, the expression of proteins involved in iron metabolism. Throughout their lives, individuals are exposed to various metals during personal and/or occupational activities. Therefore, better knowledge of the connections between iron and other metals could improve our understanding of iron-related diseases, especially the variability in phenotypic expression, as well as a variety of diseases in which iron metabolism is secondarily affected. Controlling the metabolism of other metals could represent a promising innovative therapeutic approach.

## INTRODUCTION

Iron is a metal found at very high levels in Earth’s crust that is essential for cell life due to its major role in most biological systems and metabolic pathways through its involvement in oxygen transport and delivery, and participation in a large number of enzymatic processes (Crichton, 2001a). In addition, iron may participate in the genesis of reactive oxygen species (ROS) through the Fenton and Haber–Weiss reactions (Wardman and Candeias, 1996). Production of ROS alters proteins, lipids, and DNA during oxidative stress favoring the development of cellular alterations that lead to the development of lesions in organs (Meneghini, 1997; Crichton, 2001b).

During the beginning of the third millenary, knowledge of iron metabolism has consistently progressed with the identification of numerous genes encoding iron metabolism proteins, including hepcidin, and a better understanding has been gained of systemic iron metabolism. These discoveries led to the identification of a strong potential relationship between iron and other metals, contributing to an understanding of previous unexplained findings described primarily during the 20th century.

Here, our objective is to provide evidence of true metal interactions that likely represent a way of better understanding metal metabolism and metal-related diseases in the future.

## IRON METABOLISM PLAYERS

Iron is a transition metal with a metabolism characterized by the following: (i) a major role of iron found in plasma (1–2 mg), which is delivered to every cell in the body; (ii) constant recycling of iron associated with hemoglobin within erythrocytes, followed by iron export toward plasma (20 mg/day); (iii) the absence of an active, iron excretory process, and iron losses (1–2 mg/day) are uncontrolled; and (iv) a tuning of digestive absorption that must strictly compensate for iron losses (Andrews, 1999, p. 677). Thus, most of the iron required by the organism daily (20 mg/day) is provided by the erythrophagocytosis process that occurs within macrophages.

In plasma (Andrews, 1999), iron is linked in its ferric form to transferrin, a β-globin protein of hepatocytic origin. The iron saturation of transferrin is 30–45%. Iron-transferrin is taken up by cells through transferrin receptor 1 via an endocytic process. An additional step occurs in the endocytic vesicle and involves divalent metal protein 1 (DMT1; Fleming et al., 1997, 1998; Gunshin et al., 1997), which is encoded by the SLC22a1 gene. After reduction of ferric iron by the STEAP 3 protein (Lambe et al., 2009), the iron is transferred to the cytosol, where it becomes available for cells. Plasma iron is directed mainly to erythroblasts, where it associates with heme for hemoglobin synthesis (Andrews, 1999, p. 677). In some cases, mainly during iron-overload diseases, non-transferrin-bound iron may appear in the plasma (Hershko and Peto, 1987; Brissot et al., 2012). Non-transferrin-bound iron associates mainly with low-molecular-weight molecules. This form of iron can be avidly taken up by parenchymal cells, especially hepatocytes (Brissot et al., 1985). Proteins expressed at the hepatocyte membrane level, such as ZIP14 (Liuzzi et al., 2006), could participate in this process, conferring to hepatocytes, and more globally the liver, a major role in the control of excess iron in the plasma by offering an iron-storage site.

Macrophages ensure recycling of senescent erythrocytes (Andrews, 1999). The last phase of this process involves the ferroportin protein (SLC40a1 gene), which transfers ferrous iron toward the plasma (Donovan et al., 2000; McKie et al., 2000). Next, iron is oxidized by the ceruloplasmin protein, a multicopper oxidase secreted by the liver (Osaki et al., 1966; Miyajima et al., 1987; Harris, 1995). Iron is subsequently taken in charge by transferrin (Andrews, 1999).

Iron losses are mostly related to cutaneous and digestive cell peeling, urine leakage, and regular (menstruations) or occasional bleeding.

Digestive iron absorption occurs mostly in the duodenum and involves enterocytes (Andrews, 1999). Non-heme ferric iron is taken up from the digestive lumen by DMT1 (Fleming et al., 1997, 1998; Gunshin et al., 1997) after an oxidation process that could involve Dcytb, a ferrireductase protein (McKie et al., 2001). Heme iron could be taken up from nutrients by a specific transporter. Heme carrier protein 1 is a candidate (Shayeghi et al., 2005), allowing further heme processing by heme-oxygenase 1 within enterocytes, and then iron reaches the cytoplasmic iron pool (Fleming et al., 1997, 1998; Gunshin et al., 1997). In both cases, the transfer of iron in the plasma as ferrous iron is ensured by the ferroportin protein (Donovan et al., 2000; McKie et al., 2000). Iron is then oxidized in ferric iron by hephaestin, another multicopper oxidase anchored in the cell membrane at the basolateral level (Vulpe et al., 1999; Anderson et al., 2002), and/or ceruloplasmin, allowing iron to interact with plasma transferrin and its delivery to other cells (Andrews, 1999).

## SYSTEMIC REGULATORS OF IRON METABOLISM

Regulation of systemic iron metabolism involves hepcidin, a cysteine-rich, 25 amino acid, iron-inducible peptide secreted by the liver (Nicolas et al., 2001; Park et al., 2001; Pigeon et al., 2001). Schematically, serum hepcidin may interact with ferroportin protein localized on the cell membrane of macrophages and enterocytes, inducing its internalization and degradation (Nemeth et al., 2004). The global effect is a control of iron egress from cells, avoiding increased transferrin saturation, which would expose to the appearance of non-transferrin-bound iron and to the development of parenchymal iron overload, as observed during genetic hemochromatosis (Loréal et al., 2005).

Iron is then sequestered in macrophages or enterocytes within ferritin, the iron-storage protein (Munro and Linder, 1978; Theil, 1987). In macrophages, iron is mobilized toward the plasma as required based on the plasma hepcidin level. Thus, iron can be available immediately after a decrease in hepcidin. In enterocytes, stored iron (corresponding to the classical “mucosal block”) is lost during cell peeling. This mechanism is related to the low ratio between iron originating every day from enterocytes compared to macrophages and likely occurs to control iron storage over a longer period (Andrews, 1999).

Hepcidin regulators play a critical role in the maintenance of adequate serum hepcidin levels and, therefore, in the control of serum iron and systemic iron homeostasis (Muckenthaler, 2008). Inducers of hepcidin expression by hepatocytes include excess iron (Pigeon et al., 2001) and inflammation (Nemeth et al., 2003). Increased hepcidin gene transcription related to iron involves the hemojuvelin/bone morphogenetic/SMAD (HJV/BMP/SMAD) pathway, which is activated following BMP6 over-expression (Andriopoulos et al., 2009; Meynard et al., 2009). In addition, HFE and TFR2 proteins, which are both expressed on the hepatocyte cell membrane, participate in the induction of hepcidin expression under conditions of excess iron. Although a role of increased serum transferrin saturation has been proposed (Goswami and Andrews, 2006; Muckenthaler, 2008), the molecular mechanism involved and the interaction with the HJV/BMP/SMAD pathway are not fully understood. Such regulation contributes to limiting iron excess. The control of hepcidin levels is deficient during genetic hemochromatosis related to *HFE, TFR2*, *HJV*, or *HAMP* mutation in humans, leading to iron-overload diseases (Brissot et al., 2011). Inflammation strongly increases hepcidin expression through the IL6/STAT3 pathway (Wrighting and Andrews, 2006; Pietrangelo et al., 2007; Verga Falzacappa et al., 2007), contributing to limited iron bioavailability for either growing pathogenic agents during infectious disease or oxidative stress during chronic inflammation.

Factors reducing hepcidin expression include hypoxia. Such impact of hypoxia increases serum iron and transferrin saturation, allowing intense erythropoiesis to compensate for tissue hypoxia (Muckenthaler, 2008). Whether the impact of hypoxia on *HAMP* transcription involves a stimulation of hypoxia inducible factor (HIF; Peyssonnaux et al., 2007) remains to be definitively determined. Recent arguments suggest that factors secreted during erythropoiesis could impact hepatocytes and limit hepcidin expression (Muckenthaler, 2008; Zhao et al., 2013).

Taken together, these findings put the focus on the hepcidin and ferroportin duo.

## REGULATION OF INTRACELLULAR IRON METABOLISM

Within cells, an integrated system ensures the control of total iron content and distribution. The iron responsive element (IRE) and iron regulatory protein (IRP) together control the expression of proteins encoded by mRNA exhibiting an IRE nucleotide motif localized in the 5′UTR or 3′UTR (Hentze et al., 1987, 1988). When IRPs are active, they interact with the IRE, limiting the expression of proteins, such as ferritin, which has an IRE in its 5′UTR, and stabilizing mRNA, such as transferrin receptor 1 mRNA, which displays IRE motifs in its 3′UTR. This interaction is promoted by intracellular iron deficiency, the global effect being the promotion of iron ingress into the cell and associating with proteins that require this metal to reach their full activities. Conversely, in the presence of iron excess, the decrease in IRP activities leads to increased ferritin protein expression, favoring the storage of iron in a chemically inactive form, and decreased cellular iron entry due to a strong decrease in transferrin receptor 1 mRNA expression. Together, these processes avoid the production of ROS by limiting the amount of iron available for their production.

There are other iron-related proteins coded by mRNAs containing an IRE in their 5′UTR or 3′UTR, including DMT1 and ferroportin (Gunshin et al., 1997; McKie et al., 2000).

## IRON-RELATED DISEASES CAN BE ASSOCIATED WITH ALTERATIONS IN THE METABOLISM OF OTHER METALS

Iron homeostasis is lost during iron-related diseases. Schematically, three conditions can be found: (i) true iron deficiency related to insufficient intake, malabsorption, or excessive losses (Miller, 2013); (ii) iron misdistribution linked to systemic inflammation or cell-specific processes (Ganz and Nemeth, 2009); and (iii) systemic iron excess associated with genetic iron overload (Brissot et al., 2011) or anemia related to hematological cause (Gardenghi et al., 2010), with or without transfusions. Clinical, biological, and genetic characterization of these conditions pinpoints the links between iron and other metals.

### IRON DEFICIENCY

Iron deficiency has been associated with abnormal absorption of metals from the digestive lumen. Pollack et al. (1965) reported a significant impact of iron deficiency, which was different according to etiology. In the rat, anemia consecutive to bleeding induced the absorption of manganese, cobalt, and iron. During iron deficiency related to poor iron intake, zinc absorption was increased (Pollack et al., 1965). Notably, a biological marker of chronic iron deficiency is an increase in zinc-protoporphyrin, which reflects the substitution of iron by zinc as a substrate for ferrochelatase during the last step of heme synthesis (Labbe et al., 1999). Conversely, absorption of calcium, magnesium, mercury, and copper was not significantly affected. Interestingly, supplementation of the diet with iron did not modify cobalt and manganese hyperabsorption. More recently, it was reported (Nam and Knutson, 2012) that iron status, especially iron deficiency, may increase the expression of ZIP 5, a zinc transporter, and conversely decreases the hepatic expression of ZIPs 6, 7, and 10. Moreover, iron-deficient rats had higher hepatic copper concentrations. The authors underlined that zinc transporters could play a role in hepatic iron/metal homeostasis during iron deficiency.

In particular, the relationship between iron deficiency and non-iron metals has been investigated in the brain. Iron deficiency may increase zinc concentration in the midbrain and hippocampus, whereas copper concentrations have been reported to be increased in the cerebral cortex and corpus striatus (Shukla et al., 1989). In addition, Erickson (Erikson et al., 2004) showed that iron deficiency induced an increase in manganese concentration in the putamen, globus pallidus, and substantia nigra. Zinc concentrations were also increased. The authors suggested that an increase in DMT1 expression related to iron deficiency could be involved. In the same way, iron depletion and loading increased brain manganese concentrations in young rats; this impact remained significant for 9 weeks. Moreover, the uptake of manganese by the brain, liver, kidneys, and bones was significantly increased by excess iron in younger rats. Manganese supplementation increased radioactive iron uptake by the brain, liver, and kidneys of rats receiving control and Fe-deficient diets compared to rats supplemented with dietary iron (Chua and Morgan, 1996). DMT1 could be involved. Indeed, in Belgrade rats, the DMT1 mutation similarly affected manganese and iron metabolism, suggesting that they share similar transport mechanisms in the cells of erythroid tissue, duodenal mucosa, kidneys, and the blood–brain barrier (Chua and Morgan, 1997).

Another argument for an impact of iron deficiency on the metabolism of other metals is that iron deficiency confers a susceptibility to tissue accumulation of heavy, potentially toxic, metals, such as cadmium, nickel, and lead (Six and Goyer, 1972; Flanagan et al., 1978; Tandon et al., 1993). Mechanisms may include increased digestive absorption and metal accumulation in tissues, such as has been reported for cadmium (Flanagan et al., 1978). For nickel, a dynamic study in rats argued for increased absorption and decreased excretion (Tallkvist and Tjalve, 1997).

### IRON MISDISTRIBUTION

The most frequent cause of iron misdistribution is the inflammatory process, which is the second most common etiology of anemia worldwide through the anemia of chronic disease (ACD; Weiss and Goodnough, 2005). Regarding iron metabolism, ACD is characterized by macrophagic iron sequestration and decreased iron absorption. The underlying mechanism involves increased plasma hepcidin levels, which limit the expression of ferroportin on the cell membrane (Ganz, 2011). Such situations have been associated with alterations in the metabolism of other metals. Thus, zinc and copper serum concentrations were reported to be increased in an acute model of inflammation (Milanino et al., 1986). Notably, zinc plays a major role in inflammation and the immune response, and zinc supplementation may improve innate immunity during inflammatory/infectious processes in acute, septic models (Knoell et al., 2009; Bao et al., 2010). Recently, zinc deficiency was reported to up-regulate the JAK/STAT3 pathway and could contribute to the severity of inflammation (Liu et al., 2014). The concentrations of calcium, strontium, and iron are increased in neutrophil granules, but the manganese increase in leukocytes was not localized to the granules (Hallgren et al., 1989). Whether the impact of inflammation on non-iron metals is related to decreased iron bioavailability and/or due to other independent mechanisms, including cytokine production, is not known.

Regarding heavy metals, a recent report described a role for ZIP14, which is involved in the cellular uptake of non-transferrin-bound iron and cadmium accumulation during inflammation (Min et al., 2013).

### SYSTEMIC IRON OVERLOAD

Systemic iron-overload diseases include genetic iron overload –involving mutations in iron-related genes –, and secondary iron overload associated with hematologic diseases, and iron excess associated with liver diseases.

Regarding genetic iron-overload diseases, reports emphasize disturbances in other metals. Thus, during HFE-related hemochromatosis, in which low hepcidin levels lead to an abnormal increase in both digestive iron absorption and macrophagic iron release, an increase in hepatic zinc concentration has been reported (Adams et al., 1991), whereas plasma zinc concentration was normal (Brissot et al., 1978).

Abnormalities in manganese metabolism have been reported, in addition to those reported by Chua and Morgan (1997). In a mouse model of genetic hemochromatosis, Jouihan et al. (2008) showed that mitochondrial manganese uptake was altered, leading to mitochondrial dysfunction. Moreover, Kim et al. (2013) reported that the digestive absorption of manganese was strongly increased in *Hfe*^-/-^ mouse model further emphasizing the relationship between iron and manganese metabolism.

A metal for which strong interactions with iron have been reported is cobalt. Cobalt may mimic iron deficiency by stabilizing HIF and, in turn, induce a large number of genes related to hypoxia and iron metabolism (Schuster et al., 1989; Yuan et al., 2003; Karovic et al., 2007). Cobalt may also reduce hepcidin expression by hepatocytes without involvement of the transcriptional factor HIF-1 (Braliou et al., 2008). Digestive absorption of cobalt was increased in hemochromatotic patients or patients exhibiting liver cirrhosis complicated by iron overload (Valberg et al., 1969; Olatunbosun et al., 1970). In patients with hepatic steatosis or cirrhosis and normal iron status, digestive absorption of cobalt and iron was not affected compared to controls. Conversely, in patients with cirrhosis and iron deficiency, both cobalt and iron absorption were increased to similar levels as a group of patients exhibiting iron deficiency alone.

Blood lead concentration was found to be increased during genetic hemochromatosis, in contrast with transfusional iron overload (Barton et al., 1994). Iron depletive treatment performed by phlebotomies in genetic hemochromatotic patients induced an increase in cadmium uptake (Akesson et al., 2000).

## ALTERATIONS OF NON-IRON METALS MAY IMPACT IRON METABOLISM

Numerous reports have studied the impact of modulations of non-iron metals on iron metabolism. Here, we will focus on copper, zinc, cobalt, manganese, and lead.

### COPPER

Alterations in copper metabolism may strongly affect iron metabolism. Thus, the discovery of mutations in the ceruloplasmin gene provided an explanation for the peculiar phenotype of systemic iron overload involving the brain, which contrasts low plasma iron levels and concomitant anemia (Miyajima et al., 1996; Gitlin, 1998; Loréal et al., 2002). No significant alterations in copper metabolism were found. These effects are related to the role of ceruloplasmin, a multicopper oxidase, in oxidizing ferrous iron before its transferrin linkage in plasma. This role of ferroxidase activity has been reported for many years (Osaki et al., 1966). Notably, copper metabolism is a therapeutic target during Wilson disease, which is characterized by a toxic accumulation of copper due to a defect in the ATP7B gene (Tanzi et al., 1993; Huster, 2010). Thus, zinc oral supplementation has been used to limit copper absorption. The mechanisms involved are competition between zinc and copper, as well as an induction of enterocyte metallothioneins by zinc, as they can link copper as well as zinc (Schilsky et al., 1989). However, whether zinc is associated with copper chelating therapy during the active or maintenance phase of treatment remains to be discussed (Schilsky, 2009). Recently, the impact of zinc on copper metabolism was reinforced by the description of a new form of systemic iron overload (Videt-Gibou et al., 2009) related to secondary aceruloplasminemia resulting from excessive zinc intake (Nations et al., 2008). The defect is corrected by copper supplementation.

### ZINC

Modulations in zinc metabolism may also affect iron metabolism. Thus, in swine, zinc supplementation induces liver iron depletion without modulation of hepatic copper content (Cox and Hale, 1962). In rats, zinc supplementation decreased growth and favored anemia (Cox and Harris, 1960). O’Neil-Cutting et al. (1981) demonstrated that, in rats receiving low amounts of copper, a zinc-enriched diet induced anemia and low hepatic copper concentrations. This effect was not observed in animals with a balanced zinc diet, supporting the potential impact of zinc on erythropoiesis when appropriate cofactors exist. Another study suggested that zinc supplementation during gestation and lactation could have a differential effect on liver iron content, whereas copper content is not affected (Ketcheson et al., 1969). Taken together, these reports support that the potential effects of zinc on other metals should be evaluated, despite the presence of studies supporting recommendations for zinc supplementation (Hess and King, 2009). Moreover, despite numerous publications on the impact of zinc on the brain, especially during neurodegenerative diseases in which abnormal excess levels of iron have been reported, the effect of zinc appears to be ambiguous (review in Kawahara et al., 2014). Zinc has been proposed as an antioxidant molecule to improve neurodegenerative disease. However, zinc has also been suspected to favor Alzheimer’s disease and neuronal death. Knowing the potential impact of zinc on iron metabolism, and whether this effect in neurodegenerative disorders is partly related to abnormal iron metabolism in the brain, warrants further investigation.

### COBALT

In trace amounts, cobalt is essential, as it is an integral part of the vitamin B_12_ complex and has a physiological impact on iron metabolism by contributing to erythropoiesis (Simonsen et al., 2012). In addition, cobalt supplementation may facilitate tolerance to hypobaric hypoxia (Shrivastava et al., 2008), which could be related to the modulation of HIF-regulated genes in order to promote oxygenation. Cobalt chloride supplementation has been evoked as a potential doping strategy in athletes (Lippi et al., 2005). To date, cobalt is not explicitly prohibited by world anti-doping agencies, despite its potential toxicity in the case of abnormal exposure. An increase in hemoglobin/hematocrit levels and polycythemia has been recorded in humans, rats, and dogs exposed to cobalt, demonstrating a strong impact on iron metabolism (see the US Agency for Toxic Substances and Disease Registry: http://www.atsdr.cdc.gov/toxprofiles/tp33.pdf).

### MANGANESE

Manganese, an essential component of metalloenzymes, is also essential for cell life. One of the main manganese-requiring enzymes is manganese superoxide dismutase, which plays a major role in counteracting oxidative stress, especially with iron, by detoxifying the superoxide radicals (Martinez-Finley et al., 2013). Some published data support an impact of manganese on iron metabolism. In cattle, oral manganese supplementation in animals receiving a low copper diet leads to decreased DMT1 expression in enterocytes. In addition, the down-regulation of hepcidin and ferroportin mRNA was found in the liver of animals receiving a copper-deficient diet alone (Hansen et al., 2010). In rats, during the neonatal period in animals receiving an iron-deficient diet, manganese supplementation of dams was reported to increase brain levels of manganese, chromium, zinc, cobalt, aluminum, molybdenium, and vanadium in the pups. In addition, iron decreased and copper increased in the brain (Garcia et al., 2007).

### LEAD

For many years, lead exposure was reported to strongly modulate iron metabolism. Multiple mechanisms are likely involved in this interaction. As known for a long time, excess lead inhibits δ -aminolevulinic acid dehydratase and ferrochelatase activities, in particular, induces zinc protoporphyrin accumulation in erythrocytes, and favors the occurrence of microcytic hypochromic anemia (Ku et al., 1990; Braun, 1999). In addition, lead has been reported to limit the transfer of iron from endosomes toward the cytoplasm (Qian et al., 1997). More recently, lead exposure in rats was reported to decrease serum iron and transferrin saturation levels (Moshtaghie et al., 2013). In workers exposed to lead, copper, and ceruloplasmin serum concentrations are increased, but no significant alteration in iron and zinc serum levels has been found (Kasperczyk et al., 2012). Potential interactions between lead and ceruloplasmin protein may explain a decrease in ceruloplasmin-linked ferroxidase activity (Leelakunakorn et al., 2005). *In vitro*, lead decreased transferrin synthesis in a human hepatic cell line (Barnum-Huckins et al., 1997). However, such an impact has not been reported in lead-exposed workers (Kasperczyk et al., 2012).

## MOLECULAR EVENTS AND PATHWAYS LINKING IRON METABOLISM TO NON-IRON METAL METABOLISM

### A ROLE FOR PROTEINS INVOLVED IN IRON TRANSPORT

**FIGURE 1:**
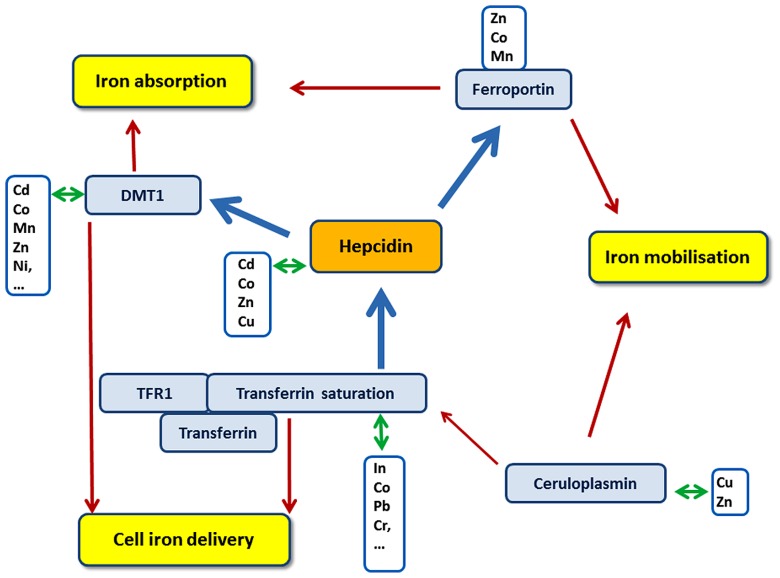
**Schematic representation of potential connections between iron, hepcidin and non-iron metals.** Special focus has been made on three major processes in iron metabolism – digestive iron absorption, iron mobilization and cell iron delivery (yellow boxes) – and some major proteins and parameters directly involved in iron metabolism (blue boxes). White boxes indicate non-iron metals for which relationships have been reported with adjacent iron metabolism protein (For details see in the text). Red arrows represent an involvement of the protein in the targeted biological process. Blue arrows indicate an impact of the protein on the expression/activity of the targeted protein.

There are proteins playing a role in the processes contributing to maintain iron homeostasis that have been associated to non-iron metal metabolisms (**Figure 1**).

DMT1 is a potential major link between iron and other divalent cations. The first description of DMT1 was associated with iron metabolism, especially in enterocytes and erythroblasts (Gunshin et al., 1997). However, DMT1 was also demonstrated to be able to take in charge other metals (Gunshin et al., 1997). Such a potential role of DMT1 with metals other than iron was reinforced by recent data showing that the efficacy of metal transport was very important for Cd^2^, Fe^2+^, Co^2+^, and Mn^2+^, and lesser for Zn^2+^, Ni^2+^, and Vo^2+^. However, the authors found that DMT1 expression did not stimulate the transport of Cr^2+^, Cr^3+^, Cu^+^, Cu^2+^, Fe^3+^, Ga^3+^, Hg^2+^, or VO^+^ (Illing et al., 2012). In addition, iron processing by DMT1 was competitively inhibited by Co^2+^ and Mn^2+^.

Ferroportin is also a candidate for interactions between iron and other metals. Ferroportin was initially reported as the iron exporter. However, recent data suggest that manganese, zinc, and cobalt could also be taken in charge by ferroportin (Troadec et al., 2010; Yin et al., 2010). This finding was confirmed by data showing that ferroportin expression in *Xenopus* oocytes enhance the efflux of ^65^Zn and ^57^Co but not ^64^Cu, ^109^Cd, or ^54^Mn. In addition, iron, zinc, and cobalt egress were inhibited by oocyte exposure to hepcidin (Mitchell et al., 2014).

Transferrin is the major plasma protein involved in iron delivery to cells. However, some data suggest that cobalt competes with iron on transferrin (Harrington, 1992). Thus, biochemical determination of non-transferrin-bound iron levels can be altered in the presence of cobalt (Gosriwatana et al., 1999). Others have suggested that transferrin may interact with manganese (Aschner and Gannon, 1994), indium, bismuth (Li et al., 1996; Zhang et al., 2004), copper (O’Neil-Cutting et al., 1981), and chromium (Harris, 1977). Transferrin has also been reported to interact with lead when present in excess (Leelakunakorn et al., 2005). In addition, when saturated by non-iron metals, transferrin could compete with iron-transferrin during its interaction with transferrin receptor 1 (Ha-Duong et al., 2008; El Hage Chahine et al., 2012). Taken together, these data suggest that metals other than iron could, by using transferrin as a Trojan horse, play a role in metal distribution within the body. This could also theoretically modulate the kinetics of iron ingress into cells. However, the impact on iron uptake is likely moderate due to the ratios of concentrations of different metals and iron in plasma and to a lower affinity of these non-iron metals for transferrin receptor 1 (El Hage Chahine et al., 2012). The low amount of non-iron metal linked to transferrin has been recently confirmed in mouse serum (Herrera et al., 2014). The same authors studying wild-type mice and transferrin-deficient mice also showed that transferrin does not play a major role in the delivery of manganese, copper, or zinc to tissues. Moreover, they suggest that an increase of tissue manganese found in transferrin deficient mice is linked to an indirect effect of transferrin deficiency on hepcidin expression or iron metabolism (Herrera et al., 2014). The interaction of transferrin with In^3+^ and Cu^2+^ has been shown to induce conformational changes similar to Fe^3+^; in addition, Al^3+^ causes a conformational change of a somewhat smaller magnitude, whereas Hf^4+^ (hafnium) does not induce significant conformational changes (Grossmann et al., 1993). Gallium transferrin is also taken in charge by the transferrin receptor (Chikh et al., 2007).

Taken together, these findings suggest that physiological or pathological modulation of the expression or activity of iron proteins, such as DMT1 or ferroportin, as well as transferrin, could modify the metabolism of other metals.

### A ROLE FOR HEPCIDIN IN THE METABOLIC CONTROL OF NON-IRON METALS

Hepcidin, the key regulator of iron bioavailability in plasma, exerts its role by controlling the expression of ferroportin protein, its main target, on the cell membrane (**Figure 1**).

Hepcidin may also modulate DMT1 expression. DMT1 expression and activity were decreased *in vitro* at the apex of Caco-2 cells after exposure of the basolateral part of the cells to hepcidin (Brasse-Lagnel et al., 2011). Notably, during genetic hemochromatosis, DMT1 and ferroportin are highly expressed in enterocytes, as observed during iron deficiency, and the inverse correlation between serum iron parameters and protein expression found in enterocytes of iron-deficient patients disappeared in genetic hemochromatosis patients (Zoller et al., 2001). Whether hepcidin plays a role on the modulation of DMT1 in addition to its impact on ferroportin has not been addressed. Molecular mechanisms could involve the proteasome (Brasse-Lagnel et al., 2011).

These findings suggest that physiological and pathological modulation of hepcidin levels in plasma could also strongly modulate metal fluxes in the body. Low levels of hepcidin, as observed during iron deficiency, genetic hemochromatosis, or liver diseases, favor the uptake of iron and, likely, non-iron metals that can be processed by DMT1 and/or ferroportin. Such mechanisms could contribute to the clinical findings reported in older (aforementioned) studies, especially those regarding cobalt (Valberg et al., 1969; Olatunbosun et al., 1970).

In addition, hepcidin expression could be regulated by non-iron metals at the transcriptional level. Cadmium, copper, and zinc could modulate hepcidin expression through interactions between the metal-responsive element located in the hepcidin promoter with metal transcription factor 1 (MTF1; Balesaria et al., 2010). In addition, the authors showed that hepcidin expression was increased by cadmium, copper, and zinc salts in HuH7 hepatoma cells and decreased by cobalt and iron salts. In addition, methallothionein-1 expression followed hepcidin modulation by non-iron metals, but not by iron *per se*.

Animals developing hemochromatosis related to the *Hfe*^-/-^ genotype in association with abnormally low hepcidin levels were recently reported to exhibit significant modulation of their digestive microbiota. Whether this observation is related to iron excess only or whether it involves a molecular defect consecutive to HFE dysfunction is not known. However, as bacteria present with differential equipment, including siderophores, for acquiring metals from the diet and different requirements regarding other metals, modulation of microbiota during iron overload could have an impact on the absorption of iron or other metals.

Finally, hepcidin has been reported to physically interact in plasma with transition metals, including copper, nickel, and zinc (Tselepis et al., 2010). Whether this observation has significant biological relevance is not currently known.

## INTERACTIONS BETWEEN IRON AND HEPCIDIN AND THE METAL CONNECTION IN IRON-RELATED DISEASES

Until now, one of the major problems in iron-related diseases has been understanding the variability of clinical and biological expression.

This is especially true in genetic hemochromatosis related to the p.Cys282Tyr mutation. The homozygous mutation is found in three subjects per thousand in the Caucasian population. However, an increase in serum transferrin saturation, which is the earliest biochemical expression of the disease, only occurs in 50% of homozygous subjects. The clinical manifestations that impact life expectancy or welfare are even rarer (Brissot et al., 2011). These discrepancies led to a search for putative genetic cofactors associated with the p.Cys282Tyr mutation. Some explanations have been proposed, including digenism or polymorphisms in other genes directly or indirectly involved in iron metabolism (Merryweather-Clarke et al., 2003; Island et al., 2009). In addition, an impact of hormonal status (Latour et al., 2014) and environmental factors related to metabolic syndrome (Desgrippes et al., 2013) has been found.

The identification of interconnections between non-iron metals and their potential impact on iron metabolism provides a new way to explore the differential expression of iron-related diseases, especially in a world where every individual may be exposed to various metals during personal and occupational life, as well as after prosthesis implantation.

## Conflict of Interest Statement

The authors declare that the research was conducted in the absence of any commercial or financial relationships that could be construed as a potential conflict of interest.
